# Associations of adverse childhood experiences with dental fear, and the mediating role of dental fear on caries experience: the Young-HUNT4 Survey

**DOI:** 10.1186/s12903-025-06486-1

**Published:** 2025-07-10

**Authors:** Lena Myran, Yi-Qian Sun, Göran Dahllöf, Tiril Willumsen, Anne Rønneberg, Audun Havnen, Therese Kvist, Abhijit Sen, Hedda Høvik

**Affiliations:** 1 Center for Oral Health Services and Research, Mid-Norway (TkMidt), Trondheim, Norway; 2 Department of Psychology, Faculty of Social and Educational Sciences, Norwegian University of Science and Technology (NTNU), Trondheim, Norway; 3 Department of Clinical and Molecular Medicine, Norwegian University of Science and Technology (NTNU), Trondheim, Norway; 4 Department of Pathology, Clinic of Laboratory Medicine, St. Olavs Hospital, Trondheim, Norway; 5 Division of Orthodontics and Pediatric Dentistry, Department of Dental Medicine, Karolinska Institutet, Stockholm, Sweden; 6 Department of Paediatric Dentistry, Behavioural Science and Forensic Dentistry, Institute of Clinical Dentistry, University of Oslo, Oslo, Norway; 7 Division of Psychiatry, Nidaros Community Mental Health Centre, St. Olavs University Hospital, Trondheim, Norway; 8 Department of Paediatric Dentistry, Dental School, The University of Western Australia, Perth, WA Australia; 9 Department of Public Health and Nursing, Faculty of Medicine and Health Sciences, Norwegian University of Science and Technology (NTNU), Trondheim, Norway

**Keywords:** Adverse childhood experience, Adolescent, Bully victimisation, Caries, Dental fear, Sex difference, Young-HUNT

## Abstract

**Background:**

Adverse childhood experiences (ACEs) may contribute to dental fear, which can negatively affect long-term oral health. We aimed to examine associations between specific and cumulative ACEs and dental fear in adolescents, investigate possible sex differences, and explore the potential mediating role of dental fear in the relationship between ACEs and caries experience.

**Methods:**

This cross-sectional study included 5882 Norwegian adolescents aged 13–17 years from the Young-HUNT4 Survey in Norway. Self-reported ACEs (i.e., physical and sexual abuse, witnessing violence, parental divorce, parental alcohol problems, and bully victimisation) and dental fear were combined with clinical measures of caries experience. Logistic regression was used to estimate odds ratios (ORs) and 95% confidence intervals (CIs) for the associations between ACEs and dental fear. Effect modification by sex was assessed using the likelihood ratio test. A counterfactual-based mediation analysis was conducted to estimate a potential mediating effect of dental fear on the relationship between ACEs and caries experience, with results presented as ratios of means (RMs) with bias-corrected 95% CIs.

**Results:**

All specific ACEs were associated with higher odds of reporting dental fear, compared to those not reporting the given ACE. Adolescents reporting any ACE had a 74% higher likelihood of reporting dental fear compared to those without ACEs (OR 1.74, 95% CI 1.29–2.33). A dose-response relationship was observed, with a one-unit increase in ACE exposure associated with higher odds of reporting dental fear (OR 1.25, 95% CI 1.15–1.36). There was evidence of effect modification by sex, with the associations between any ACE and dental fear being more pronounced in females. Further, dental fear accounted for 5.9% of the total effect in the association between any ACE and caries experience.

**Conclusions:**

Specific and cumulative ACEs were associated with dental fear among adolescents, with a stronger association in females. Our findings indicate a partial mediating role of dental fear in the association between ACEs and caries experience. This study highlights the value of incorporating ACEs and dental fear into patient assessment.

**Supplementary Information:**

The online version contains supplementary material available at 10.1186/s12903-025-06486-1.

## Introduction

Adverse childhood experiences (ACEs) are potentially traumatic events that children or adolescents are exposed to before the age of 18, such as physical, sexual, and emotional abuse, bully victimisation, neglect, and household dysfunction. From a life-course perspective, ACEs are major contributors to a wide range of health problems and have been linked to premature mortality in adults [1]. Furthermore, multiple ACEs demonstrate a graded relationship with both physical and mental health problems, where the risk increases with increasing exposure to ACEs [1, 2]. Research has consistently reported that exposure to ACEs is associated with mental disorders, including anxiety, depression, and suicidality [3]. Pertinent to our study, ACEs have been linked to dental fear [4], although the underlying mechanisms remain unclear and the evidence is still inconclusive [5].

Dental fear refers to an unpleasant emotional reaction to threatening stimuli related to dental treatment. Dental anxiety is a more generalised fear experienced by dental patients [6]. Dental phobia involves an extreme fear of dental stimuli that patients actively avoid or endure with intense fear and is classified as a specific phobia in the American Psychiatric Association's Diagnostic and Statistical Manual of Mental Disorders (DSM-5) [7]. Dental fear, dental anxiety, and dental phobia represent varying degrees of the same psychological condition [8]. In this study, we use the term “dental fear” to encompass all these degrees.

The prevalence of dental fear varies from 13.3% to 29.3%, and sex differences have been reported, with females exhibiting a higher prevalence than males [6]. The aetiology of dental fear is commonly categorised into endogenous (internal) and exogenous (external) sources [8]. Among exogenous factors, Oosterink et al. identified experiencing helplessness and pain during childhood dental treatment as major contributing risk factors [9]. However, traumatic events occurring outside the dental clinic, such as sexual abuse [10], interpersonal violence, and bully victimisation [11], have also been associated with dental fear in adolescents. Regardless of origin, dental fear often leads to avoidance of treatment, creating a self-sustaining cycle of deteriorating dental health and increased fear, which further reinforces avoidance [12].

Dental caries, the most prevalent chronic disease worldwide, poses a public health concern [13]. While epidemiological studies on caries in children are common, fewer focus on adolescents [14], despite a peak in caries prevalence among those aged 15–19 years and young adults [13]. Adolescence thus represents a critical window for oral health, as this period is marked by increased autonomy that may affect oral hygiene and dietary habits. Studies have also identified an association between childhood adversities and increased caries in both children and adolescents [15].

Although caries is both preventable and manageable, the role of psychological risk factors such as dental fear has received limited attention. Dental fear may contribute to the avoidance of dental care [12], thereby increasing the risk for untreated caries. Previous studies have identified an association between dental fear and increased dental treatment needs in younger populations [16, 17]. More broadly, anxiety-related conditions have been shown to mediate the association between ACEs and health outcomes such as substance abuse disorders [18], irritable bowel syndrome [19], and somatic complaints in trauma survivors [20]. Building on these findings, we hypothesise that dental fear may serve as a mediating pathway between ACEs and caries experience.

Studies investigating the associations between ACEs, dental fear, and caries experience in adolescent populations are scarce. Most of these studies focus on only one or two specific types of ACEs [10] or lack clinical measures of caries experience [11]. Further, while associations between ACEs and caries experience in younger populations have been identified [21], the potential mediating role of dental fear in this relationship remains unexplored. A mediating effect of dental fear supports targeted interventions aimed at reducing dental fear and improving oral health in adolescents with a history of ACEs.

The aim of this study was to assess associations between ACEs, dental fear, and caries experience in Norwegian adolescents. We hypothesised that dental fear in adolescents was associated with specific ACEs and expected to find a dose-response relationship between ACEs and increased likelihood of reporting dental fear. Given a higher prevalence of dental fear among females, we also explored whether the association between ACEs and dental fear was modified by sex. Finally, we aimed to assess the potential mediation effect of dental fear on the associations between ACEs and caries experience.

## Methods

### Study population

We used data from the fourth wave of the Trøndelag Health Study (HUNT4), a population-based survey in Norway. All adolescents (*n* = 10 608) in the northern part of Trøndelag County aged 13–19 years were invited to participate in the Young-HUNT4 Survey [22]. Data were collected at schools between August 2017 and January 2019, during which participants completed web-based questionnaires in a classroom setting and were interviewed by trained nurses [22]. Adolescents not enrolled in the school system were invited to participate at alternative sites such as apprenticeship seminars, public outreach services, and at HUNT4 study sites. These adolescents received a letter of invitation and questionnaire by regular mail.

**Fig. 1:**
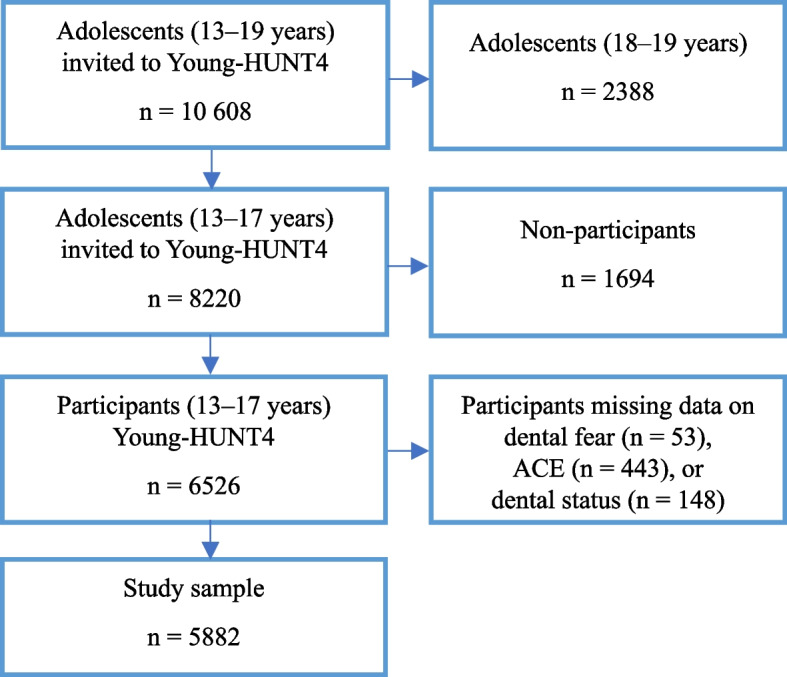
Flowchart of the study population

Data on caries experience were obtained from dental records from the Public Dental Service (PDS) in Trøndelag County. Children in Norway receive free dental care in the PDS until age 19, with almost all children (97.5%) attending regular oral health examinations [23]. Of the 10 608 adolescents invited in Young-HUNT4, 8220 were 13–17 years old and within the age definition of ACEs. In total, 6526 adolescents (response rate 79.4%) accepted the invitation. Of these, 53 were excluded due to missing data on dental fear, 443 due to missing data on one or more ACEs, and 148 had no dental status. The final sample consisted of 5882 adolescents, representing our study population (Fig. 1).

### Exposure variables

Data on ACEs were obtained from the Young-HUNT4 questionnaire and included eight specific types of adversity, covering experiences of physical abuse, witnessing violence, sexual abuse, parental divorce, parental alcohol problems, and bully victimisation. Questions and answer options for these variables have previously been presented [21].

Five exposure variables were based on the University of California at Los Angeles Post-traumatic Stress Disorder (UCLA PTSD) Reaction Index [24] and included sexual abuse by a peer, sexual abuse by an adult, physical abuse by someone close, physical abuse by others, and being a witness to violence. These were used as binary variables: exposed (once / several times) or not exposed to the specific ACE.

Parental divorce was registered for participants whose parents were divorced or separated and included cases where the parents had moved back together after being separated for more than a year.

Parental alcohol problems were defined as an ACE if participants reported having seen either of their parents intoxicated monthly or more often.

Bully victimisation was assessed using four questions based on the Olweus Bully/Victim Questionnaire and Slonje and Smith [25], covering verbal, physical, and digital bullying, as well as social exclusion. Responses were recorded using a 4-point Likert scale (1: never, 2: 1–3 times a month, 3: once a week, 4: almost every day). A bully score was calculated by summing the responses from all four questions (range: 4–20) and defined as an ACE if the score was 6 or more. Bully victimisation was also used as a continuous variable, to assess the possible dose-response relationship of bully victimisation on dental fear.

We also aggregated the specific ACEs (range 0–8) into an ACE score to assess both experiencing one or more ACEs (i.e., any ACE) and the dose-response relationship of exposure to multiple adversities.

### Outcome and mediator variables

Dental fear was assessed using the Dental Anxiety Question (DAQ): “Are you afraid of going to the dentist?” [26]. The level of anxiety was recorded on a 4-point Likert scale and dichotomised into dental fear (very afraid / afraid) and no dental fear (a bit afraid / not afraid). DAQ has been validated both in child and adult populations [26–28].

Caries data were extracted from electronic dental records (Opus Dental) provided by the PDS in Trøndelag County. For caries diagnostics the PDS in Norway uses a five-grade classification where grades 1 and 2 constitute enamel caries and grades 3–5 caries at dentin level [29]. The diagnoses were based on radiographic and clinical examinations [30]. Caries experience at dentine level was further recorded using the Decayed (caries grade 3–5), Missing and Filled Teeth (DMFT) index, which reflects the cumulative burden of caries. We used participant dental status summaries from 2018 to match the Young-HUNT4 period (August 2017–January 2019); if not available, the dental status from 2017 or 2019 was used. In preparation for the Young-HUNT4 data collection, PDS personnel in Trøndelag participated in a one-day training seminar featuring theoretical instructions on caries classification and group-based training annotating bitewing radiographs. Annotated results were reviewed in plenary, although inter-rater reliability was not assessed. Additionally, the PDS in Trøndelag has since 2013 conducted caries diagnostic seminars for dentists and dental hygienists. Further details are provided in Myran et al. [21].

**Fig. 2:**
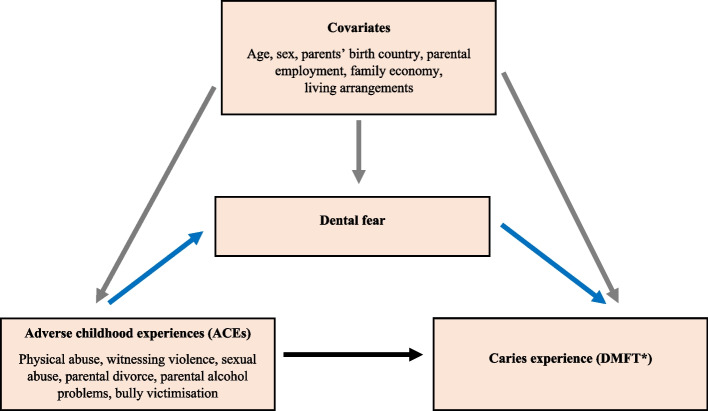
Conceptual model of the association between adverse childhood experiences (ACEs) and caries experience (DMFT) partially mediated by dental fear. The blue-coloured arrows indicate the potential mediation effect of dental fear. The grey-coloured arrows indicate confounding variables. The black-coloured arrow indicates the direct effect of the association between ACEs and caries experience. *DMFT: Decayed, Missing and Filled Teeth

In the present study, dental fear was also included as a potential mediator in the association between ACEs and caries experience. Figure 2 presents the conceptual model of this relationship.

### Covariates

Covariates included age (continuous), sex (female or male), parental birth country, socioeconomic factors, and living arrangements. Parents’ birth country was categorised based on whether both, one, or neither parent was born in a Nordic country. If data were missing for one parent (*n* = 69), the response for this parent was coded as non-Nordic-born. Socioeconomic factors included parental employment and perceived family economy. Parental employment was categorised based on whether both, one, or neither parent was employed. If data were missing for one parent (*n* = 158), the response for this parent was coded as not employed. Family economy was based on the adolescent’s self-reported assessment relative to others and dichotomised as adequate (better / same as most others) or worse. Living arrangements were categorised as living with both parents, both parents shared, one parent, or not living with parents (i.e., with other guardians, alone, in dormitories, or institutions).

### Statistical analyses

All statistical analyses were performed using Stata v18 (StataCorp, College Station, Texas, USA). To assess the association between ACEs and dental fear, we used logistic regression analyses, reporting odds ratios (ORs) with 95% confidence intervals (CIs). The models were adjusted for potential confounders including age, sex, parents’ birth country, parental employment, family economy, and living arrangements. The likelihood ratio test was used to assess whether sex modified the association between ACEs and dental fear.

We used counterfactual-based mediation analysis [31] to estimate the mediation effect of dental fear on the association between ACEs and caries experience. This approach allowed estimation of the natural direct effect (NDE) from ACEs on caries experience, the natural indirect effect (NIE) through dental fear as the mediator, and the total effect (TE). Only adolescents aged 16–17 years were included in the mediation analysis. This selection was based on our previous study using the same data, in which we found an association between ACEs and caries experience among adolescents aged 16–17, but not among those aged 13–15 [21]. The analysis was performed using the *paramed* command, combining logistic regression models for the mediator (dental fear) and negative binomial regression models for the outcome variable DMFT. NDE, NIE, and TE were estimated and presented as ratios of means (RMs) with bias-corrected 95% CIs, using bootstrap resampling (*n* = 500). The proportion mediated was calculated by dividing the natural-log-transformed NIE by the natural-log-transformed TE. The models were adjusted for the same confounders; however, the parents’ birth country, parental employment, and living arrangements were recoded as binary variables to accommodate programming requirements.

## Results

This study included a general adolescent population of 5882 participants, 51.1% female, from the Young-HUNT4 Survey, Norway. In total, 5.4% reported dental fear, with a higher prevalence among 16–17-year-olds (6.6%) compared to 13–15-year-olds (4.5%). As shown in Table 1, 3194 (54.3%) had experienced at least one ACE (i.e., any ACE), with a higher proportion among those aged 16–17 compared to those aged 13–15. Adolescents with any ACE more often reported not living with both parents, experiencing a worse financial situation, and having parents who were unemployed, compared to those with no ACEs.

**Table 1:** Characteristics of the total study population, stratified by no ACE and any ACE (1–8 ACEs), *n* = 5882

Variables	Total sample	No ACE	Any ACE
	*n* = 5882	*n *= 2688	*n *= 3194
	n (%)	n (%)	n (%)
**Age**			
13–15 years	3335 (56.7)	1633 (60.8)	1702 (53.3)
16–17 years	2547 (43.3)	1055 (39.3)	1492 (46.7)
**Sex**			
Males	2876 (48.9)	1315 (48.9)	1561 (48.9)
Females	3006 (51.1)	1373 (51.1)	1633 (51.1)
**Nordic birth country parents**			
Both parents	5238 (89.1)	2412 (89.7)	2826 (88.5)
One parent	299 (5.1)	121 (4.5)	178 (5.6)
None of the parents	323 (5.5)	140 (5.2)	183 (5.7)
Unknown/missing	22 (0.4)	15 (0.6)	7 (0.2)
**Parental employment**			
Both parents	4355 (74.0)	2180 (81.1)	2175 (68.1)
One parent	1093 (18.6)	374 (13.9)	719 (22.5)
None of the parents	164 (2.8)	37 (1.4)	127 (4.0)
Unknown/missing	270 (4.6)	97 (3.6)	173 (5.4)
**Family economy**			
Better/same financial situation	5413 (92.0)	2592 (96.4)	2821 (88.3)
Worse financial situation	433 (7.4)	82 (3.1)	351 (11.0)
Unknown/missing	36 (0.6)	14 (0.5)	22 (0.7)
**Living arrangements**			
Both parents	3633 (61.8)	2465 (91.7)	1168 (36.6)
Both parents, but shared	1037 (17.6)	7 (0.3)	1030 (32.3)
One of the parents	716 (12.2)	43 (1.6)	673 (21.1)
Not living with parents	234 (4.0)	80 (3.0)	154 (4.8)
Unknown/missing	262 (4.5)	93 (3.5)	169 (5.3)

*ACE *Adverse childhood experience

As presented in Table 2, exposure to physical abuse by others, witnessing violence, sexual abuse by peers, parental divorce or separation, and bully victimisation were associated with 35–93% increased odds of reporting dental fear compared to those not exposed to the specific ACE. There was an association of sexual abuse by an adult, physical abuse by someone close, and parental alcohol problems with dental fear, however these associations did not reach statistical significance. Additionally, a one-unit increase in the bully score was associated with increased odds of reporting dental fear (OR 1.16, 95% CI 1.11–1.22).

**Table 2:** Associations between specific ACEs and dental fear, *n* = 5882

ACEs		Prevalence of dental fear	Crude	Adjusted^a^
	n	n (%)	OR (95%CI)	OR (95%CI)
Physical abuse, close				
No	5613	290 (5.2)	reference	reference
Yes	269	27 (10.0)	2.05 (1.35–3.10)	1.43 (0.93–2.22)
Physical abuse, other				
No	5559	286 (5.1)	reference	reference
Yes	323	31 (9.6)	1.96 (1.33–2.89)	1.77 (1.18–2.66)
Witness to violence				
No	5033	257 (5.1)	reference	reference
Yes	849	60 (7.1)	1.41 (1.06–1.89)	1.39 (1.03–1.88)
Sexual abuse, peer				
No	5513	272 (4.9)	reference	reference
Yes	369	45 (12.2)	2.68 (1.91–3.74)	1.64 (1.15–2.34)
Sexual abuse, adult				
No	5721	296 (5.2)	reference	reference
Yes	161	21 (13.0)	2.75 (1.71–4.41)	1.51 (0.92–2.48)
Parental divorce				
No	3816	173 (4.5)	reference	reference
Yes	2066	144 (7.0)	1.58 (1.26–1.98)	1.35 (1.07–1.72)^b^
Parental alcohol problems				
No	5650	295 (5.2)	reference	reference
Yes	232	22 (9.5)	1.90 (1.21–3.00)	1.48 (0.92–2.37)
Bully victimisation				
No	4909	228 (4.6)	reference	reference
Yes	973	89 (9.2)	2.07 (1.60–2.67)	1.93 (1.48–2.51)
Bully victimisation score				
One-unit increase	5882	317 (5.4)	1.18 (1.13–1.24)	1.16 (1.11–1.22)

*ACEs* Adverse childhood experiences, *OR* Odds ratio, *CI* Confidence interval

^a^Adjusted for age (continuous), sex (male/female), Nordic birth country parents, family economy, parental employment, and living arrangements

^b^Not adjusted for living arrangements

Table 3 presents the associations between exposure to multiple ACEs and dental fear, for the total sample and stratified by sex. In the total sample, experiencing any ACE was associated with higher likelihood of dental fear (OR 1.74, 95% CI 1.29–2.33) compared to those without ACE. A one-unit additional ACE increased the odds of reporting dental fear by 24% (OR 1.24, 95% CI 1.14–1.35). Furthermore, dental fear was more prevalent among females (8.0%) than males (2.7%). The likelihood ratio test indicated that the relationship between ACEs and dental fear was modified by sex (*p*-value = 0.06). In females, exposure to any ACE was associated with a 93% higher odds of reporting dental fear (OR 1.93, 95% CI 1.36–2.74).

**Table 3:** Associations of cumulative ACEs with dental fear, in the total sample and stratified by sex, *n *= 5882

		**Prevalence of dental fear**	**Crude**	**Adjusted**^**a**^
	n (%)	n (%)	OR (95% CI)	OR (95% CI)
**Total sample**				
No ACE	2688 (45.7)	98 (3.7)	reference	reference
Any ACE (1–8 ACEs)	3194 (54.3)	219 (6.9)	1.95 (1.52–2.48)	1.74 (1.29–2.33)
One-unit ACE increase	5882	317 (5.4)	1.35 (1.25–1.46)	1.24 (1.14–1.35)
**Stratified by sex**				
**Females**				
No ACE	1373 (45.7)	68 (5.0)	reference	reference
Any ACE (1–8 ACEs)	1633 (54.3)	172 (10.5)	2.26 (1.69–3.02)	1.93 (1.36–2.74)
One-unit ACE increase	3006	240 (8.0)	1.39 (1.27–1.51)	1.29 (1.16–1.42)
**Males**				
No ACE	1315 (45.7)	30 (2.3)	reference	reference
Any ACE (1–8 ACEs)	1561 (54.3)	47 (3.0)	1.33 (0.84–2.11)	1.37 (0.80–2.34)
One-unit ACE increase	2876	77 (2.7)	1.14 (0.95–1.37)	1.11 (0.91–1.35)

*ACEs *Adverse childhood experiences, *OR* Odds ratio, *CI* Confidence interval

^a^Adjusted for age (continuous), sex (male/female), Nordic birth country parents, family economy, parental employment, and living arrangements

Through the mediation pathway as modelled in Fig. 2, we assessed the indirect effect of dental fear on the associations between ACEs and caries experience in 16–17-year-olds. Table 4 presents the mediation analysis results, indicating that dental fear explained 5.9% of the total effect in the association between exposure to ACEs and caries experience. For one-unit increase in ACE, the indirect effect through dental fear accounted for 8.6% of the total effect.

**Table 4:** The direct, indirect by dental fear, and total effect of ACEs on caries experience (DMFT) in 16–17-year-olds, *n* = 2547

	Natural direct effect (NDE)	Natural indirect effect (NIE)	Total effect (TE)	Proportion mediated^a^
	RM (95% CI)	RM (95% CI)	RM (95% CI)	
Any ACE (1–8 ACEs)^b^				
Crude	1.33 (1.22–1.47)	1.03 (1.01–1.04)	1.36 (1.24–1.52)	
Adjusted^c^	1.31 (1.17–1.47)	1.02 (1.00–1.03)	1.33 (1.19–1.51)	5.9%
One-unit ACE increase				
Crude	1.10 (1.06–1.14)	1.01 (1.01–1.02)	1.11 (1.08–1.15)	
Adjusted^c^	1.09 (1.05–1.12)	1.01 (1.00–1.01)	1.09 (1.06–1.13)	8.6%

*ACEs* Adverse childhood experiences, *DMFT* Decayed, Missing, and Filled teeth, *RM* Ratio of means, *CI* Confidence interval

^a^Proportion mediated: ln RM_NIE_/ln RM_TE_

^b^Reference: no ACE

^c^Adjusted for age (continuous), sex (male/female), Nordic birth country parents, family economy, parental employment, and living arrangements

## Discussion

In this Norwegian population-based cross-sectional study of 5882 adolescents aged 13–17 years, 54.3% reported exposure to at least one ACE, and 5.4% reported dental fear. All specific ACEs were associated with higher odds of reporting dental fear, compared to those not exposed to the given ACE. Additionally, there was evidence of a dose-response relationship where the accumulation of adversities increased the likelihood of dental fear. Sex modified the associations between ACEs and dental fear, with the association being more pronounced in females. Mediation analysis indicated that dental fear partially mediated the associations between ACEs and caries experience among the older adolescents.

In our study population, 5.4% reported dental fear, a lower prevalence compared to studies among adolescents from Sweden (8.2%) [11], Norway (12.0%) [32], and Finland (17.2%) [33]. The lower prevalence may be explained by methodological differences in how dental fear was assessed. While the Swedish study reported a slightly higher prevalence, the prevalence in the Finnish and Norwegian studies were more than twice as high. The latter two studies employed multi-item instruments such as Corah´s Dental Anxiety Scale (DAS) and the Modified Dental Anxiety Scale (MDAS), which may be more sensitive in detecting mild or subclinical levels of dental fear than the single-item measure and dichotomous categorisation used in our study. Furthermore, older adolescents in our sample reported a higher prevalence of dental fear (6.6%) than younger adolescents (4.5%), which contrasts with most comparable prevalence studies in adolescent populations [6]. However, some studies have found a higher prevalence of dental fear in older children compared to younger [34, 35], and in adults compared to adolescents [36].

Adolescents exposed to any one of the included specific ACEs showed increased odds of reporting dental fear compared to those with no experience of the given ACE, although not all results were statistically significant. Bully victimisation was associated with higher odds of reporting dental fear, consistent with studies among Swedish adolescents [11] and Brazilian schoolchildren [37]. We also found a dose-response relationship between cumulative bully victimisation and dental fear, consistent with Evans et al. who reported increased rates of anxiety among adolescents who experienced persistent bullying [38]. In contrast to our findings, Noirrit-Esclassan et al. did not find a dose-response relationship between bully victimisation and dental fear specifically [11]. Furthermore, our findings are in accordance with studies in younger populations exploring associations between dental fear and sexual abuse [10], witnessing violence [11], and parental divorce [39]. Among the adolescents in our study, reporting parental alcohol problems was associated with increased odds of dental fear. This aligns with studies reporting a higher likelihood of anxiety disorders in adolescents exposed to parental alcohol problems [40]. Furthermore, we found an association between physical abuse and higher odds of dental fear. This contrasts with findings from a study on Swedish adolescents [11]. However, the question measuring physical violence in the Swedish study was phrased with a different level of specificity than ours, which may partly explain the differing results.

Our finding that exposure to multiple ACEs was associated with higher odds of reporting dental fear aligns with the cumulative risk model and is consistent with a study identifying a graded relationship between ACEs and anxiety, depression, and behavioural problems in adolescents [41]. For dental fear specifically, our findings parallel those of Noirrit-Esclassan et al., who reported that the adjusted odds ratio for dental fear increased from 1.4 with one type of adversity to 1.9 with two types of adversity [11].

In our study, there was a higher prevalence of dental fear among females, consistent with studies on dental fear in younger populations [11, 16, 42]. In sex-stratified analyses, the association between exposure to ACEs and increased odds of dental fear was more pronounced among females. These findings align with studies reporting sex differences in dental fear among Brazilian schoolchildren who had experienced bullying [37] and Nigerian adolescents who had experienced sexual abuse [10]. Studies in adult populations have also reported similar relationships, with increased dental fear among Dutch female patients exposed to sexual assault [43], and heightened fear of shame in the dental setting among Norwegian female patients with abuse histories [44]. Taken together, these findings highlight sex-specific vulnerabilities and underscore the need for tailored approaches in dental care.

Mediation analysis supported that dental fear had a slight indirect effect on the relationship between ACEs and caries experience in the 16–17-year-olds. Previous studies have demonstrated that fear-driven dental avoidance was associated with increased dental treatment needs [45], while interventions that reduced dental fear were associated with more regular dental visits in adults [46]. In our model, dental fear mediated 5.9% of the total effect suggesting that this is only one of several pathways involved. Caries develops through a complex interplay of biological, psychological, and socio-environmental factors including oral hygiene habits, dietary habits, self-esteem and social support, and access to dental care [47–51]. Although dental fear may explain only a part of the association between ACEs and caries, our findings highlight the importance of preventing and treating dental fear, particularly among vulnerable adolescents with a history of adversity.

Several mechanisms may explain the association between ACEs and dental fear. Traumatic events can shape psychological responses, such as heightened apprehension [52], cognitive difficulties [53], and emotional dysregulation [54]. In dental settings, features such as lying flat, opening one's mouth, or having an authority figure lean over can trigger memories of past traumatic events, a phenomenon known as trauma coupling [55], increasing the fear of dental visits [56]. Interpersonal traumas, including bullying or abuse, are also associated with increased interpersonal sensitivity [57] and a heightened sense of distrust and social threat [58]. Given the relational and intimate nature of dental treatment, dental procedures may be perceived as invasive and beyond one's control [56]. The dental situation can trigger feelings of shame, guilt and inferiority [12], threat [56], and embarrassment [59]. Experiencing these emotions during dental treatment may lead to exhaustion or flashbacks, which can both increase and maintain dental fear [56].

Our findings have clinical implications for both dental professionals and clinical psychologists. In our study, a history of ACEs was associated with increased odds of reporting dental fear, and prior studies have linked dental fear to increased caries experience in younger populations [16, 17]. Dental professionals should routinely inquire about adverse experiences to identify and support vulnerable individuals, while clinical psychologists should consider both trauma history and oral health, as individuals with significant dental fear may avoid dental health services altogether. Given that increased interpersonal sensitivity and lack of trust are common long-term consequences of childhood adversity [57, 58], an empathic, person-centred approach is essential in all care settings. Creating a safe and supportive environment is key to building trust and enabling effective dental care. However, such environments require systemic support for sustained dentist-patient relationships. Potential financial and time constraints in dental care systems, including the lack of reimbursement for individualised, time-intensive care, limit the feasibility of tailored interventions. Introducing dedicated reimbursement schemes, facilitating individualised treatment, may benefit both patients with dental fear and those at risk of becoming fearful. Additional strategies include oral health programmes that empower adolescents to communicate effectively with dental professionals, thereby strengthening therapeutic alliances. Finally, funding interdisciplinary care models, where dentists and psychologists collaborate to treat dentally anxious patients may reduce dental fear among those with a history of ACEs, ultimately reducing oral health disparities. In Norway such models have been implemented for both children [60] and adults [61, 62].

Strengths of this study include a population-based design, large sample size, high response rate, and the integration of self-reported data from adolescents with clinical measures from dental records. However, the interpretation of our results should consider certain limitations. Not all types of childhood adversities were assessed, and different types of adversities may have varying impacts; experiencing abuse may have a different impact than experiencing parental divorce [63]. Our analysis did not account for the timing, duration, or co-occurrence of adversities, nor did we adjust for current mental health status, a factor previously associated with dental fear [64]. Other unmeasured variables such as parental income and education may also have caused residual confounding. Retrospective self-reports of ACEs may be subject to recall bias, potentially leading to underreporting. However, a high reliability between documented childhood maltreatment and later self-reports from adolescents has been reported [65]. In our study we used the single-item Dental Anxiety Question (DAQ), available in Young-HUNT4, to assess dental fear. The DAQ is less used and not as comprehensive compared to other available instruments. However, DAQ was reported to correlate with more complex instruments [26, 27] and is considered a valid instrument in epidemiological research when the use of a longer questionnaire is not practical [66]. Additionally, although the overall response rate among participants was high, participation among adolescents not attending school was low [22], possibly underrepresenting those with more physical and mental health problems. Among those excluded from the study sample, the proportions having neither parent born in a Nordic country, experiencing parental unemployment, and not living with both parents were slightly higher compared to those in the study sample (Supplementary Table 1). Furthermore, caries data extracted from dental records may be subject to variations due to multiple examinators. An attempt to address this issue was made by implementing a one-day training seminar on caries diagnostics. Finally, due to the cross-sectional design, we cannot infer causal relationships between ACEs and dental fear, nor determine temporal sequencing in the mediation analysis.

## Conclusion

This study examined associations between exposure to ACEs and dental fear among adolescents aged 13–17 years. Several specific ACEs were associated with a higher likelihood of reporting dental fear compared to those not exposed to the given ACE. Exposure to multiple ACEs was associated with higher odds of reporting dental fear, with the odds gradually increasing with the number of adversities experienced. The association between exposure to ACEs and dental fear was more pronounced in females. Furthermore, dental fear partially mediated the association between childhood adversity and caries experience among the older adolescents. These findings highlight the potential value of dental practitioners addressing both ACEs and dental fear in all elements of dental care, and the benefit of clinical psychologists considering oral health in their patient assessments. To build on these insights, future longitudinal and interventional studies are needed.

## Supplementary Information

Supplementary Material 1.

## Data Availability

The data are stored in HUNT databank. HUNT Research Centre has permission from the Norwegian Data Protection Authority to store and handle these data. To protect participants’ privacy, HUNT Research Centre aims to limit storage of data outside HUNT databank and cannot deposit data in open repositories. HUNT databank holds precise information on all data exported to different projects and can reproduce these on request. There are no restrictions regarding data export given approval of applications to HUNT Research Centre. For more information see: www.ntnu.edu/hunt/data. Inquiries regarding access to data are directed to: kontakt@hunt.ntnu.no.

## Abbreviations

ACE: Adverse childhood experience
CI: Confidence interval
DAS: Corah´s Dental Anxiety Scale
DAQ: Dental Anxiety Question
DMFT: Decayed, Missing and Filled Teeth
HUNT: The Trøndelag Health Study
MDAS: Modified Dental Anxiety Scale
NIE: Natural indirect effect
NDE: Natural direct effect
OR: Odds ratio
PDS: Public Dental Service
RM: Ratio of means
TE: Total effect
UCLA PTSD Reaction Index: The University of California at Los Angeles Post-traumatic Stress Disorder Reaction Index
Young-HUNT4 Survey: The adolescent component of the HUNT Study
