# Supplementary material for: Associations of adverse childhood experiences with dental fear, and the mediating role of dental fear on caries experience: the Young-HUNT4 Survey
Source: BMC Oral Health. 2025 Jul 10;25:1141. doi: 10.1186/s12903-025-06486-1 (PMC12247215; doi:10.1186/s12903-025-06486-1)
Supplement: Supplementary file 1 — Supplementary Material 1. [file 12903_2025_6486_MOESM1_ESM.docx]

**Supplementary Table 1.** Characteristics of the study sample (n = 5882) and excluded participants (n = 644)

| **Variables** | **Study sample**  n = 5882  n (%) | **Excluded participants**  n = 644  n (%) |
| --- | --- | --- |
|  |  |  |
| **Age** |  |  |
| 13–15 years | 3335 (56.7) | 384 (59.6) |
| 16–17 years | 2547 (43.3) | 260 (40.4) |
|  |  |  |
| **Sex** |  |  |
| Males | 2876 (48.9) | 316 (49.1) |
| Females | 3006 (51.1) | 328 (50.9) |
|  |  |  |
| **Nordic birth country parents** |  |  |
| Both parents | 5238 (89.1) | 482 (74.8) |
| One parent | 299 (5.1) | 40 (6.2) |
| None of the parents | 323 (5.5) | 97 (15.1) |
| Unknown/missing | 22 (0.4) | 25 (3.9) |
|  |  |  |
| **Parental employment** |  |  |
| Both parents | 4355 (74.0) | 402 (62.4) |
| One parent | 1093 (18.6) | 138 (21.4) |
| None of the parents | 164 (2.8) | 42 (6.5) |
| Unknown/missing | 270 (4.6) | 62 (9.6) |
|  |  |  |
| **Family economy** |  |  |
| Better/same financial situation | 5413 (92.0) | 538 (83.5) |
| Worse financial situation | 433 (7.4) | 46 (7.1) |
| Unknown/missing | 36 (0.6) | 60 (9.3) |
|  |  |  |
| **Living arrangements** |  |  |
| Both parents | 3633 (61.8) | 334 (51.9) |
| Both parents, but shared | 1037 (17.6) | 100 (15.5) |
| One of the parents | 716 (12.2) | 116 (18.0) |
| Not living with parents | 234 (4.0) | 41 (6.4) |
| Unknown/missing | 262 (4.5) | 53 (8.2) |
|  |  |  |

Data are given as number of participants (column percentage)
